# Carotid Stump Syndrome: An Uncommon Cause of Recurrent Ipsilateral Strokes

**DOI:** 10.7759/cureus.12688

**Published:** 2021-01-13

**Authors:** Vishnu Nagalapuram, Cindrel Tharumia Jagadeesan, Balakrishnan Ramasamy

**Affiliations:** 1 Internal Medicine, The University of Alabama at Birmingham (UAB) School of Medicine, Montgomery, USA; 2 Internal Medicine, Creighton University, St.Joseph's Hospital and Medical Center, Phoenix, USA; 3 Neurology, PSG Institute of Medical Sciences & Research, Coimbatore, IND

**Keywords:** carotid stump, recurrent stroke

## Abstract

Carotid stump syndrome is a rare cause of recurrent cerebrovascular accidents. Carotid stump is the patent proximal remnant below the completely occluded internal carotid artery (ICA). Cerebral and retinal ischemic symptoms seen after complete occlusion of ipsilateral ICA is known as carotid stump syndrome. Known for causing recurrent ipsilateral cerebrovascular accidents, it is a potentially treatable entity. The therapeutic goal is medical management with a statin, dual anti-platelet therapy along with surgical intervention either with an endovascular repair or carotid endarterectomy. Herein, we present a case of carotid stump syndrome managed medically.

## Introduction

Complete occlusion of the internal carotid artery (ICA) with a patent proximal remnant of it, which can be demonstrated by carotid duplex scan, computed tomography angiography, or magnetic resonance angiography, is called carotids stump [1]. Carotid stump syndrome (CSS) is defined as the persistence of cerebral or retinal ischemic symptoms after complete occlusion of the ipsilateral ICA [2]. CSS is one of the recognized causes of recurrent ipsilateral cerebrovascular accidents (CVAs) after complete occlusion of the ipsilateral ICA. Both hemodynamic and embolic factors have been attributed to the recurrent transient ischemic attacks (TIAs) and CVAs [3, 4]. Therapeutically, surgical exclusion of the ICA stump combined with endarterectomy of the ipsilateral external carotid artery (ECA) and endovascular revascularization of the occluded ICA have been successful. Only a handful number of cases have been reported from South Asian countries. Herein, we present a case with one such rare entity.

## Case presentation

A 64-year-old obese gentleman with a known past medical history of essential hypertension, moderate aortic stenosis, also a chronic smoker and an alcoholic, presented with right ataxic hemiparesis and dysarthria. Computed tomography (CT) of the brain showed a left parietal lobe infarct. Carotid and vertebral artery Doppler showed thrombotic occlusion of the left ICA with the absent flow. CT angiogram showed complete occlusion of left ICA with a carotid stump. The patient was started on a statin and antiplatelet therapy - aspirin. Stump excision was offered; however, the patient and family opted to proceed with medical management. The patient underwent physical and occupational therapy along with medical management, resulting in symptomatic improvement. He was discharged on a statin and single antiplatelet therapy.

**Figure 1:**
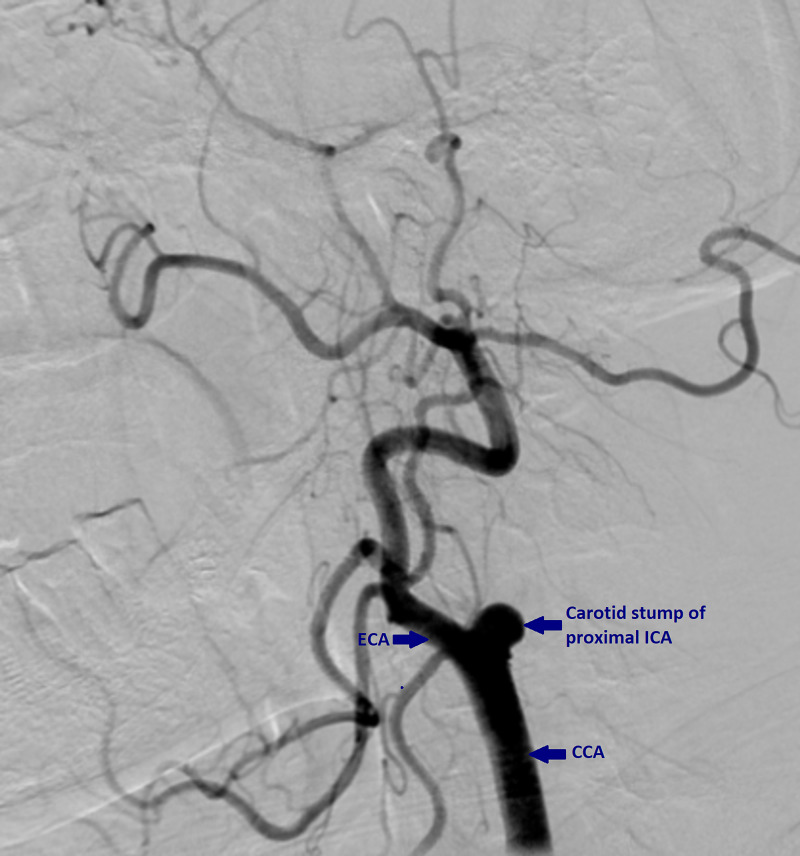
An image from digital subtraction angiography showing the stump formed due to complete occlusion of ICA ICA - internal carotid artery; ECA - external carotid artery; CCA - common carotid artery

Seven months following the initial episode, the patient again presented with right hemiparesis and dysarthria. During the interval period, the patient had six episodes of TIAs characterized by motor aphasia. Magnetic resonance imaging (MRI) of the brain showed acute on chronic left parietal lobe infarct in the left middle cerebral artery territory and complete occlusion of the left ICA with the carotid stump. Digital subtraction angiography showed complete occlusion of the left ICA and reformation of the supra-clinoid segment of the left ICA and anterior and middle cerebral arteries through collaterals and flow across the circle of Willis (Figure 1). After excluding other embolic sources with comprehensive workup, including negative coagulopathy tests, negative echocardiography with bubble study, and negative 24 hour-Holter monitoring, the diagnosis of CSS was made. Since the patient was unwilling to undergo stump excision, he was medically managed with dual antiplatelet therapy with aspirin, dipyridamole, and high dose statin. The patient did not receive thrombolytic therapy during any of these episodes due to financial reasons. Five months follow up period did not show any recurrence of symptoms.

## Discussion

Theoretically, complete occlusion of the ICA should eliminate the incidence of most anterior circulation embolic strokes. Due to the patent proximal carotid stump, there is turbulent flow and hence the formation of thrombus as a result of platelet-fibrin aggregation. Hemodynamic and embolic factors attributing towards the persistence of cerebral or retinal symptoms have been explained by the hemodynamic failure in the occluded ICA that leads to cerebral hypo-perfusion and intermittent micro-embolization from the thrombotic carotid stump to brain, respectively [3, 4]. These micro-emboli find their path to the brain tissue commonly via the patent external carotid-internal carotid anastomosis with the reverse flow in the ophthalmic artery in the presence of complete occlusion of the ipsilateral ICA. Although most of the cases reported had symptoms related to this pathway, our patient’s TIAs and CVAs did not include any visual symptoms. Other possible sources of micro-emboli from the extracranial vascular system like ipsilateral common carotid artery (CCA) or ECA; the carotid bifurcation via a patent ECA, contralateral carotid vessels via the circle of Willis, distal thrombus occluding the ipsilateral ICA or from the distal tail of the thrombus in the occluded ICA have been reported [5]. Hence, before making a CSS diagnosis, all other possible sources of emboli should be excluded, including cardiac sources and atheromatous lesions in the aortic arch.

Advocated treatment modalities include stump excision with or without endarterectomy of the ipsilateral ECA as described by Kumar et al. and endovascular excision with wall stenting across the stenosis with or without post stenting balloon angioplasty [2, 6-8]. ECA endarterectomy or post stenting balloon angioplasty should be considered when there is complete occlusion of ipsilateral ICA with atherosclerosis or stenosis of the ECA, respectively. Also, risk factors of thromboembolic events should be closely monitored and controlled to prevent the recurrence of symptoms. Most reported cases were managed by risk factor modification, medical management with antiplatelet therapy and statin, and the option of surgical or endovascular intervention. In our case, since the patient was unwilling to undergo the stump excision, we managed him medically with combination antiplatelet therapy with aspirin, dipyridamole, and high dose statin. Since most of the cases reported so far were individual case reports or case series, there is no evidence to recommend one treatment modality over the other. Offering anticoagulation for these patients should further be explored given the embolic nature of this pathology.

## Conclusions

Although uncommon, CSS is a potentially treatable cause of recurrent cerebrovascular accidents. CSS should be considered in patients with recurrent TIAs and CVAs. Patients should be offered surgical or endovascular treatment whenever possible, along with medical therapy.
